# Linkages between poverty and food insecurity in Pakistan: Evidence from urban and rural households in Peshawar

**DOI:** 10.12669/pjms.39.2.7122

**Published:** 2023

**Authors:** Zeeshan Kibria, Muhammad Naseem Khan, Saima Aleem, Zia ul Haq

**Affiliations:** 1 Dr. Zeeshan Kibria, Additional Director ORIC, Khyber Medical University, Peshawar, Pakistan; 2 Dr. Muhammad Naseem Khan, Associate Professor Population Medicine, College of Medicine, Qatar University, Qatar; 3 Dr. Saima Aleem, Senior Research Fellow, Khyber Medical University, Peshawar, Pakistan; 4 Dr. Zia ul Haq, Vice Chancellor / Professor of Public Health, Khyber Medical University, Peshawar, Pakistan

**Keywords:** Poverty, Dietary Diversity, Food Security, WASH, Households

## Abstract

**Objective:**

To assess household poverty, sanitation and hygiene practices, and food security in both urban and rural settlements of district Peshawar.

**Methods:**

We conducted this cross-sectional study from March 2019 to October 2019 in the urban and rural households of Peshawar, KPK. Using stratified random sampling, 554 households (HH) having children and young adolescents of age 5-19 years, adult men > 19 – 62 years, and adult women >19 - 62 years were included in this study. Data was collected using comprehensive tool comprised of all validated questionnaires and was analyzed using SPSS Version 24.0

**Result:**

Within the urban clusters, the maximum number of households (n=29) were from Gari Baloch and the minimum number of households (n=7) were from Gulberg. In the rural clusters, the maximum number of households surveyed (n=41) were from Lamara, minimum(n=21) was from Chargula. The average age of household heads was 44.5 ±12.5 with mean age slightly higher in urban areas (45.1 ±11.8) compared to 44.0 ±13.2 in rural areas. The mean poverty score was 56.8 (±11.6) with 72.1% non-poor households, and 94.2% households being food secure. Handwashing practices were highly prevalent among all the HH, however, handwashing practices before eating were comparatively lower in all HH (45.2%), lowest (37.8%) among rural households.

**Conclusion:**

The findings of the study revealed both non-poor and food secure households with satisfactory water, hygiene and sanitation practices.

## INTRODUCTION

Globally, the subject of the burden of poverty and food security had been attention-seeking and extensively investigated. Malnutrition, emerging as a consequence of both the concepts is highly prevalent in the majority of the low- and middle-income countries (LMICs) as a major public health concern.^1^ Both axes of malnutrition, under – and over-nutrition are equally detrimental. It’s a double-edged sword as any individual’s physical and intellectual activity is hindered by malnutrition whereas overnutrition is a direct contributor to many diseases.^2^

Food security and malnutrition both are interlinked to poverty, social and health related inequalities and poor hygiene practices each fueling the other to a life threatening level creating an interdigitated complexes with their trajectories being shaped or hampered by diverse factors ranging from dietary, biological, and behavioral factors to socioeconomic, environmental, and sociocultural factors.^3^

Pakistan had been in the line of fire for all the poverty, nutrition and malnutrition-related issues of public health concern^4^, exacerbated by diverse factors diverting the focus of humanitarian agencies and key players from much needed nutritional supplementation and fortification interventions across Pakistan. Food security, in Pakistan, has emerged as a challenge resulting from spurt of urbanization and population growth. Despite being an agricultural dominant country, food insecurity still prevails, a calamity evident from the 2018 National Nutritional Survey of Pakistan, according to which 4/10 children under five years of age are stunted while 17.7% suffer from wasting. At the provincial or regional level, stunting is highly prevalent (48.3%) in Khyber Pakhtunkhwa-Newly Merged Districts, KP-NMD (previously known as Federally Administered Tribal Areas, FATA) as compared to 40 % in KP.^5^ During the fiscal year 2019-20, a survey conducted by the Pakistan Social and Living Standards Measurement, out of 100 households, 16.4 reported moderate to severe food insecurity.^6^ Even in households having good purchasing power, the family members tend to appear to have food insecurity.^6^

In Pakistan, the household, being the basic economic and financial unit, determines the nutritional status of residing members, so the concept of malnutrition, food security, dietary diversity, and poverty is woven around this unit and interlinked with household size, maternal educational status, purchasing power, food supply, climate and healthcare.^7^,^8^

Research in Pakistan is mostly diverted to children, adolescents, and women of reproductive age in a nutritional context. Based on the poverty and food insecurity there is a definite disconnect at the local level regarding lack of quality data in Khyber Pakhtunkhwa, encompassing household head, adult male, adult female, and child as a household entity, providing a subjective base to research at provincial household level.

This paper reports the baseline characteristics of the households with the assessment of household poverty, sanitation and hygiene practices and food security in both urban and rural settlements of district Peshawar.

## METHODS

We conducted a cross-sectional survey in the urban and rural households of Peshawar, KP from March to October 2019. Ethical approval was obtained from Khyber Medical University (DIR/KMU-EB/MC/000553).

### Study Population & Sample Frame:

The sample size was calculated using openepi, considering the 60% proportion of food insecurity in households^9^, 95% confidence level and 5% margin of error and design effect 1.0, and attrition rate of 50% considering the polio infodemic in Peshawar^10^, the sample size was 554. After refusals, 521 households were included in the final study and analysis.

Respondents were selected using stratified random sampling. Block wise distribution (urban & rural) of Peshawar based on the number of households, was obtained from the Pakistan Bureau of Statistics.

Enumeration blocks, (ten villages’ councils, VC) from rural and (ten neighborhood councils, NC) from urban areas were randomly selected from the sampling frame. Line listing was performed for all the randomly selected subunits based on the map which revealed a range of 150 – 400 households in the selected clusters.

SPSS Version-24 was used to select 50 households from each cluster keeping in view a 50% rejection rate. From the list of selected households, one household was randomly selected for the starting point in the said cluster. The household head was contacted and after written informed consent, data was collected.

### Inclusion criteria:

- Households having children and young adolescents of age 5-19 years.
- Adult men > 19 – 62 years, adult women >19 - 62 years were included in this study.

### Exclusion Criteria:

- Household members already beneficiaries of any nutrition-related behavioral or clinical intervention/program and households with any member with under/postgraduate education in nutrition sciences were also excluded.

The data collection tools used in this study were derived from validated questionnaires. The demographic component of the questionnaire (age, marital status, educational status, occupation of household head, number of adult males, adult females and children in the HH) was adapted from Household Integrated Economic Survey (HIES) 2018-19.^11^

Socioeconomic component was adopted from Pakistan Poverty Scorecard.^12^ Pakistan poverty scorecard has cut-offs/ score ranges as extremely/ultra-poor (0-11), chronically poor (12-18), transitory poor (19-23) and transitory vulnerable (24-34), transitory non poor (35-50), non-poor (51-100). Food insecurity measurement was adapted from the Household Food Insecurity Access Scale (HFIAS) to estimate the prevalence of food insecurity.^13^ Household water, sanitation, and hygiene (WASH) was adapted from the WHO/UNICEF joint monitoring program (WASH).^14^

A group of eight experienced data collectors were designated to conduct the survey. The group was trained on the questionnaire and each data collector was asked to pilot the questionnaire with at least one household and report issues to resolve during training. Each team in the field comprised of two members (one male & one female) to facilitate in interviewing.

Data was analyzed using SPSS Version-24. Before the data analysis, the data was cross checked for all the missing values and information. Baseline socio-demographic characteristics are presented and frequencies and percentages. All the variables of interest in this study were analyzed and presented in total based upon gender and urban/rural stratification of respondents.

## RESULTS

This cross-sectional study was conducted in district Peshawar inclusive of both the rural and urban areas. Out of the 521 households surveyed in Peshawar, 256 households were located in urban areas and 265 were in rural areas.

The cluster wise breakdown of the household included in the study is presented in Table-I. Within the urban clusters, the maximum households (n=29) were from Gari Baloch and the minimum number of households (n=7) were from Gulberg. In the rural clusters, the maximum number of households surveyed (n=41) were from Lamara, minimum(n=21) was from Chargula.

**Table-I:** Cluster distribution.

Urban		Rural	

Cluster name	No of HHs surveyed	Cluster Name	No of HHs surveyed
Bahadur Kallay	25	Darmangai	25
Gari Baloch	29	Masho Gaggar	25
Gulbahar	21	Mian Gujjar	25
Pakha Ghulam	25	Phandu Road	25
Peshtakhara	25	Sherkira	25
Tehkal	25	Takhtabad	25
Wazir Bagh	26	Urmar Payan	25
Taj Abad	22	Larama	41
Munshi Mohallah	25	Nahaqai	28
Gulberg	7	Chargula	21
Bilal Line Town	26	-	-

TOTAL	256	TOTAL	265

Mean household head age was calculated based upon the responses from 497HH out of 521HH. The average age of household head was 44.5 ±12.5 with mean age slightly higher in urban areas (45.1 ±11.8) compared to 44.0 ±13.2 in rural areas (Table-II).

**Table-II:** Household baseline characteristics of the study sample.

Characteristic	Total n (%)	Urban n (%)	Rural n (%)
HH head age (mean ± SD) n = 497	44.6 (12.8%)	45.2 (12.1%)	44.1 (13.4%)
20-40 years	199 (38.2%)	99 (38.7%)	100 (37.7%)
> 40-60 years	246 (47.2%)	123 (48%)	123 (46.4%)
> 60-80 years	76 (14.6%)	34 (13.3%)	42 (15.8%)
***Marital status (n = 507)***			
Unmarried	33 (6.5%)	15 (6%)	18 (7%)
Married	474 (93.5%)	235 (94%)	239 (93%)
***Occupation of HH head (507)***			
Unemployed	123 (24.3%)	63 (25.2%)	60 (23.3%)
Salaried / Pensioner / Retired	87 (17.2%)	48 (19.2%)	39 (15.2%)
Self Employed	297 (58.6%)	139 (55.6%)	158 (61.5%)
Years of schooling HH head (n = 495)	9.4 (3.9)	9.6 (3.9)	9.3 (3.9)
Illiterate	29 (5.9%)	14 (5.8%)	15 (5.9%)
Primary (1-5 years)	54 (10.9%)	24 (10%)	30 (11.8%)
Secondary (> 5-10 years)	254 (51.3%)	122 (50.6%)	132 (52%)
Higher (> 10 years)	158 (31.9%)	81 (33.6%)	77 (30.3%)
No of males in HH (n = 504)	3.1 (2.1%)	3.0 (1.8%)	3.2 (2.3%)
No of females in HH (n = 505)	2.9 (1.9%)	2.8 (1.8%)	2.9 (1.9%)
No of under 5 children in HH (n = 505)	2.2 (2.0%)	2.1 (1.9%)	2.3 (2.0%)
Mean No of children / young adolescents 5-19 years HH (n = 503)	3.1 ±2.0	3.2 ±2.1	3.0 ±1.9
***Water Hygiene & Sanitation***			
Main source of drinking water (n = 465)			
Water connection to HH	265 (57%)	138 (61.1%)	127 (53.1%)
Public tap	25 (5.4%)	16 (7.1%)	9 (3.9%)
Hand pump/well inside HH	175 (37.6%)	72 (31.9%)	103 (43.1%)
Efforts to make drinking water safe (n = 502)	33 (6.3%)	22 (8.9%)	11 (4.3%)
***No of latrines in HH (n = 521)***			
NIL	6 (1.2%)	3 (1.2%)	3 (1.1%)
1-3	464 (89.1%)	226 (88.3%)	238 (89.8%)
> 3	51 (9.8%)	27 (10.5%)	24 (9.1%)
Latrines used by others (not HH members) n = 496	172 (34.7%)	75 (30.7%)	97 (38.5%)
Soap available for handwash (observed) n = 485	425 (87.6%)	216 (90%)	209 (85.3%)
***Handwashing practices***			
Before preparing food (n = 500)	444 (88.8%)	211 (85.8%)	233 (91.7%)
Before eating (n = 498)	225 (45.2%)	129 (52.9%)	96 (37.8%)
After using latrine (n = 500)	498 (99.6%)	245 (99.6%)	253 (99.6%)
After coming home from outside (n = 499)	470 (94.2%)	227 (92.3%)	243 (96%)

The average number of males in the households (n=504) was 3.1±2.1, females (n=505HH) was 2.9±1.9, children under five years of age in HH (n=505) was 2.2±2.0 and for children and young adolescents of age 5-19 years was 3.1±1.9.

Out of a total of 521 HH, the mean poverty score of 487 households was 56.8 (±11.6) with 72.1% non-poor households. Household food insecurity was calculated from a total of 521 households with 94.2% HH being food secure (Table-III).

**Table-III:** Poverty and food security status of the household.

Characteristic	Total n (%)	Urban n (%)	Rural n (%)
Poverty score (mean SD) n = 487	56.8 (11.6)	56.9 (11.2)	56.7 (12.0)
Transitory vulnerable (24-34)	25 (5.1%)	8 (3.4%)	17 (6.8%)
Transitory non poor (35-50)	111 (22.8%)	59 (25%)	52 (20.7%)
Non poor (51-100)	351 (72.1%)	169 (71.6%)	182 (72.5%)
***HH food insecurity (n = 521)***			
Insecure food in HH	30 (5.8%)	16 (6.2%)	14 (5.3%)
Secure food in HH	491 (94.2%)	240 (93.8%)	251 (94.7%)

A major source of drinking water among households (n=465) was water connection (57%) as presented in Table-II. The proportion of urban households using handpumps/well inside the household was 31.9% compared to 41.3% in rural households. Public tap was only available in 3.9% of rural households. A very small proportion (6.3%) made efforts to make drinking water safe.

Out of 521 households, 88.3% urban and 89.8% rural HH had 1-3 latrines, however >3 latrines were available in only 10.5% of the urban HH. Handwashing practices were highly prevalent among all the HH, however, handwashing practices before eating was comparatively lower in all HH (45.2%), lowest (37.8%) among rural households.

## DISCUSSION

This study assessed the baseline characteristics, poverty scores, food security, and WASH practices of urban and rural households and based on the findings, the majority of the households were non-poor, food secure, with satisfactory WASH practices.

As per the results of present study, mean number of HH males, females, and under five children among rural households were higher as compared to urban households. For older children and young adolescents, urban households had a higher mean number compared to rural HH. Findings from a survey previously conducted in KPK encompassing the Swat and Lower Dir reported to have an overall household mean number for household members to be 8.57 and 8.68 for Swat and Lower Dir respectively. This high value of mean was attributed to the fact that joint family systems still prevail in the rural KPK in Pakistan.^15^

Poverty had always been a major indicator of sociodemographic and socioeconomic status of any household. Health related quality of life, nutritional status, malnutrition and chronic disease are all directly related to poverty in a broader spectrum. Study conducted in Indonesia about multidimensional poverty dynamic stated that “the poverty line is often; derive from consumption level of every individual and costs of the basket of the basic needs”.^16^ In our study, the mean poverty score was 56.8 (±11.6) with 72.1% non-poor households. Similar poverty score card had previously been used in the survey conducted by Pakistan Poverty Alleviation Fund (PPAF) in 2012 where Battagram district was selected to study the poverty based on poverty scorecard and as per the data mentioned in their report, approx. 54.6% households were in the range of 24-100 on poverty scorecard.^12^,^17^ In 2018, Baluchistan Rural and Community Empowerment Programme published report regarding analysis of Households Poverty Scorecard Census Data from Rural Baluchistan. According to their findings, district Washuk was at the top in terms of having 68% households falling in 0-23 poverty band and remaining 32% were in 24-100 band.^18^ Findings of another study conducted reveal that poverty decreases with increase in the age of household head, but poverty increases with increase in the size of household.^19^

Interlinked to the nexus of poverty is household food insecurity; another key variable of current study. The findings of our study project that 93.8.% of the urban households were food secure and only 6.2% were food insecure which is in contradiction to study conducted in India inducting 309 eligible household and reported 77.2% of the urban households were food insecure.^20^ Another similar study included 799 households for household food insecurity analysis in adolescents reported half of adolescent being food insecure.^21^ Occasionally, it is assumed that food insecurity at the household level is also the reflection of individual level of food insecurity. It is possible to be malnourished even while living in the food secure households due to chronic diseases, mental and physical health issues or inadequate distribution of food within individual of same household. Literature does identify the factors including ethnicity, parents and household heads age, their educational level, occupation, household size and household income to be associated with household food insecurity.^22^ The social and cultural enigma is also a contributing factor to household food insecurity apart from quality of food, its availability and accessibility.^23^,^24^

Water, hygiene and sanitation practices undeniably hold substantial value in disease prevention and healthy life by controlling spread of infectious diseases.^25^ However, in many lower-and-middle-income countries, lack of access to safe water results in poor hygiene and sanitation practices. Considering this universal fact, our study reports water, hand hygiene and sanitation practices in households under study. As per the finding of our study, water connection to household was main source of drinking water in 57% of HHs. The findings from study conducted in Sindh and KPK mentioned that in most of the households in both provinces, water is supplied via groundwater and half of the rural households had access to drinking water via handpumps, and main source of drinking water in KPK was tap water.^26^

Sanitation and latrines utilization holds significance in controlling communicable diseases, environmental pathogens, diarrhea and resulting micronutrient deficiencies.^27^ It is one of the key determinants of controlling communicable diseases although at household levels different types and forms of latrine facilities exist. According to the findings of our study, only 1.2% household had no latrine which was in line with findings of study conducted in rural Bangladesh.^28^ In terms of hand washing practices after using latrines, the prevalence in our study was 99.6% which was contradictory from another study where 85.02% households had no handwashing facility with latrines and almost 94.44% households had no handwashing facility at all within household^29^, which can be attributed to socio-cultural influences and lack of education.

### Limitations of Study:

The cross-sectional design of this research limits the establishment and formulation of causal relationship.

## CONCLUSION

The present study projects non-poor, food secure households having satisfactory WASH practices establishing linkage between poverty and food security. In future, establishing community and household level counseling programs can impart knowledge about the importance of food security linked to balance diet and healthy eating practices.

### Authors` Contribution:

**ZK** Concept development, data collection, data entry, data analysis, integrity of research.

**MNK** Tools development and validation, first draft of manuscript.

**SA** Literature search, writing final draft of manuscript and responsible for the accuracy or of the study.

**ZH** Concept development, proof reading and final approval of manuscript.

## References

[1] Caleyachetty R, Thomas GN, Kengne AP, Echouffo-Tcheugui JB, Schilsky S, Khodabocus J (2018). The double burden of malnutrition among adolescents: analysis of data from the Global School-Based Student Health and Health Behavior in School-Aged Children surveys in 57 low- and middle-income countries. Am J Clin Nutr.

[2] Anik AI, Rahman MM, Rahman MM, Tareque MI, Khan MN, Alam MM (2019). Double burden of malnutrition at household level: A comparative study among Bangladesh, Nepal, Pakistan, and Myanmar. PLoS One.

[3] Tarro L, Solà R Social and Economic Factors and Malnutrition or the Risk of Malnutrition in the Elderly: A Systematic Review and Meta-Analysis of Observational Studies. Nutrients.

[4] Asim M, Nawaz Y (2018). Child Malnutrition in Pakistan: Evidence from Literature. Children (Basel).

[6] PAPER DN (2021). Food insecurity.

[7] Akbar M, Niaz R, Amjad M (2020). Determinants of households'food insecurity with severity dimensions in Pakistan: Varying estimates using partial proportional odds model. Health Soc Care Community.

[8] Nadeem S, Khatoon A, Rashid S, Ali F (2022). Dietary Intake patterns in women with GDM and Non-GDM: A comparative study. Pak J Med Sci.

[9] Pakistan GO (2014). Pakistan Vision 2025.

[10] Dawn News (2019). Capital's polio vaccination drive suffers after Peshawar incident April 24.

[11] Statistics PBo Household Integrated Economic Survey (HIES) 2018-19 2020.

[12] Pakistan Poverty Alleviation Fund (2012). Assessment of Measuring Impact of PPAF Interventions using Pakistan Poverty Scorecard.

[13] Coates J, Swindale A, Bilinsky P (2007). Food and Nutrition Technical Assistance Project (FANTA): Household Food Insecurity Access Scale (HFIAS) for Measurement of Food Access: Indicator Guide (v3).

[14] UNICEF (2018). Core questions on drinking water, sanitation and hygiene for household surveys: 2018 update.

[15] Shahbaz B, Shah QA, Suleri A, Kazmi MA, Commins S (2014). Surveying livelihoods, service deliveryIranand governance: baseline evidence from Pakistan.

[16] Wardhana D (2010). Multidimensional poverty Dynamics in Indonesia (1993-2007). School of Economics and University of Nottingham.

[17] Fund PPA (2012). Assessment of Measuring Impact of PPAF Interventions using Pakistan Poverty Scorecard.

[18] Kakar A (2018). Analysis of Households Poverty Scorecard Census Data from Rural Balochistan;Balochistan Rural Development and Community Empowerment (BRACE)Programme. Rural Support Programme Network (RSPN).

[19] Iqbal N, Awan MS Determinants of urban poverty: The case of medium sized city in Pakistan. The Pakistan Development Review.

[20] Chinnakali P, Upadhyay RP, Shokeen D, Singh K, Kaur M, Singh AK (2014). Prevalence of household-level food insecurity and its determinants in an urban resettlement colony in north India. J Health Popul Nutr.

[21] Sheikh S, Iqbal R, Qureshi R, Azam I, Barolia R (2020). Adolescent food insecurity in rural Sindh, Pakistan: a cross-sectional survey. BMC nutrition.

[22] Shone M, Demissie T, Yohannes B, Yohannis M (2017). Household food insecurity and associated factors in West Abaya district, Southern Ethiopia 2015. Agric Food Secur.

[23] Olum S, Okello-Uma I, Tumuhimbise GA, Taylor D, Ongeng D (2017). The relationship between cultural norms and food security in the Karamoja sub-region of Uganda. J Food Nutr Res.

[24] Bikesh T, Suraj B, GC A (2020). Cultural and Social Enigmas: Missing Pieces of Food Security. J Food Nutr Res.

[25] Howard G, Bartram J, Williams A, Overbo A, Geere JA, Organization WH (2020). Domestic water quantity, service level and health.

[26] Junaid MM (2016). Water, Sanitation and Hygiene (WASH): A case study on Pakistan, in UNDP (ed.). Development Advocate Pakistan.

[27] Legge H, Halliday KE, Kepha S, Mcharo C, Witek-McManus SS, El-Busaidy H (2021). Patterns and Drivers of Household Sanitation Access and Sustainability in Kwale County, Kenya. Environ Sci Technol.

[28] Huda TMN, Schmidt W-P, Pickering AJ, Mahmud ZH, Islam MS, Rahman MS (2018). A cross sectional study of the association between sanitation type and fecal contamination of the household environment in rural Bangladesh. Am J Trop Med Hyg.

[29] Berhe AA, Aregay AD, Abreha AA, Aregay AB, Gebretsadik AW, Negash DZ (2020). Knowledge, Attitude, and Practices on Water, Sanitation, and Hygiene among Rural Residents in Tigray Region, Northern Ethiopia. J Environ Public Health.

